# Neonatal spinal muscular atrophy with brain magnetic resonance imaging hypersignal: a case report

**DOI:** 10.3389/fped.2025.1508565

**Published:** 2025-04-29

**Authors:** Xiaolin Jieda, Chaoge Yang, Yue Wu, Rong Zhang, Wanting Xu, Wenbin Dong

**Affiliations:** ^1^ Department of Neonatology, Children’s Medical Center, The Affiliated Hospital of Southwest Medical University, Luzhou, Sichuan, China; ^2^ Department of Pediatrics, Southwest Medical University, Luzhou, China; ^3^ Department of Neurosurgery, The Affiliated Hospital of Southwest Medical University, Luzhou, China

**Keywords:** neonatal, spinal muscular atrophy (SMA), SMN, brain MRI, case report

## Abstract

Spinal muscular atrophy (SMA) is an autosomal recessive genetic disorder marked by progressive, symmetrical muscle weakness and atrophy. While only a limited number of studies on human SMA have demonstrated brain involvement, there are also few reports detailing early brain MRI changes in SMA patients. In this paper, we present the case of a child whose initial symptom was limb hypotonia. The child's brain MRI revealed abnormal signal changes and genetic testing ultimately confirmed the diagnosis of SMA. By reviewing relevant literature, we aim to summarize the brain MRI signal changes observed in SMA patients and explore their possible mechanisms, with the goal of enhancing clinicians' ability to identify and treat neonatal SMA at an early stage.

## Introduction

SMA is a degenerative neuromuscular disease affecting lower motor neurons in the anterior horn of the spinal cord, primarily caused by a homozygous deletion of SMN1 on chromosome 5q13 (1, 2). Its incidence ranges from 1/6,000 to 1/10,000, with a carrier frequency of 1/40 to 1/60 (1, 2). This case initially presented with decreased muscle tone in the extremities. Although some structures of the brain MRI showed abnormal signal changes similar to bilirubin encephalopathy, clinical manifestations and additional examinations did not support this diagnosis, leading to a final diagnosis of SMA through genetic testing. Characteristic brain imaging findings in neonatal SMA are rarely reported and are often confused with conditions like bilirubin encephalopathy, hypoxic-ischemic encephalopathy, and hypoglycemic encephalopathy, making diagnosis challenging. Further research is needed to clarify the diagnostic value of brain imaging changes in SMA.

## Case report

**Figure 1:**
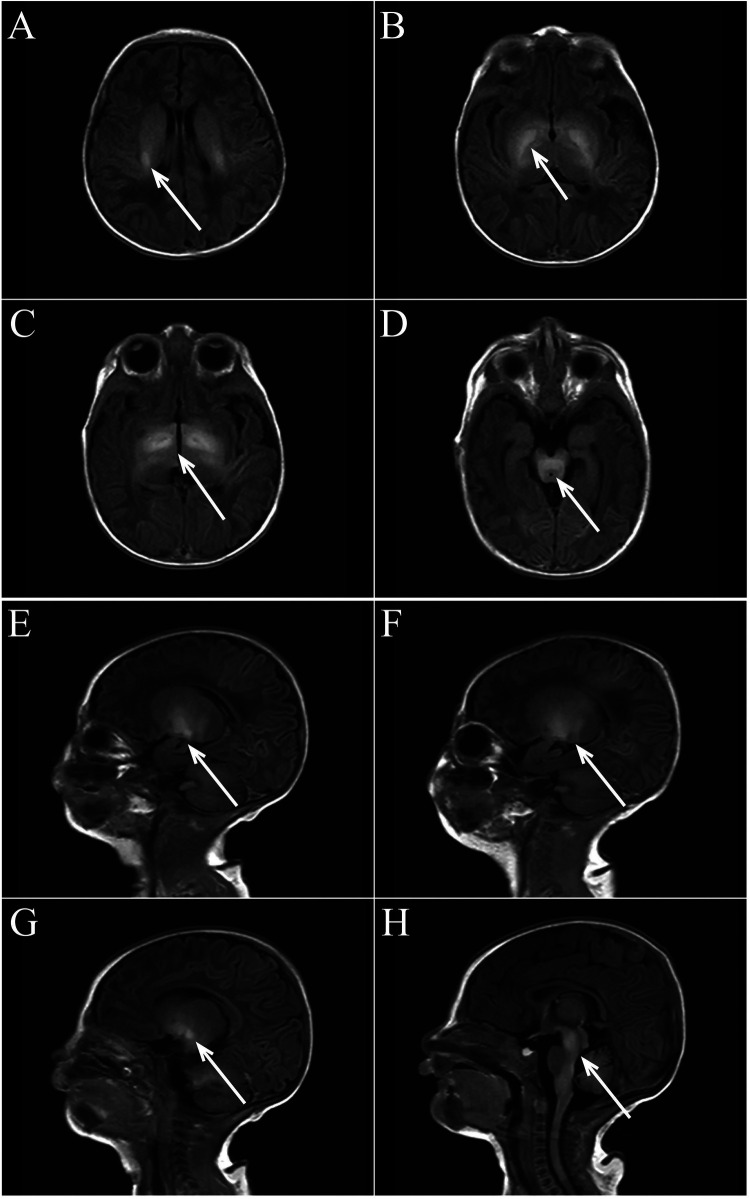
**(A–D** axial view**)** bilateral basal ganglia, thalamus, lateral ventricle, brain stem symmetrical patchy T1WI high signal shadow. **(E–H** sagittal view**)** Bilateral basal ganglia, thalamus, lateral ventricle, brain stem symmetrical patchy T1WI high signal shadow.

We report a neonate presenting with hypotonia one day after birth. Born at 40^+5^ weeks with Apgar scores of 1–5–10 min are all rated at 10 and a weight of 2,700 g, the child showed no abnormalities in the amniotic fluid, umbilical cord, or placenta, nor signs of intrauterine distress or premature rupture of membranes. The child did not show any decrease in fetal movement or fetal heart rate during pregnancy. The mother's gestational age was 32 years. His parents had no history of smoking, alcohol, drug use, or inherited metabolic diseases. The child exhibited good development, alertness, and mild jaundice. Physical examination showed no abnormalities in the heart, lungs, or abdomen and no barrel chest or paradoxical breathing exercises. The child's upper limbs could move horizontally but could not be lifted off the bed, both lower limbs could be lifted slightly off the bed but could not resist resistance, the limbs were floppy and could not be flexed naturally in the supine position, and they were in a “frog position” in the prone position. None of the tendon reflexes were elicited, the feeding and sucking reflex could be elicited, whereas the grip and hug reflexes were markedly diminished. He had difficulty in erecting his head, and he had no myoclonus, tongue spasms, or finger contractures. The “Medical Research Council Scale for Muscle Strength” suggests grade 2 muscle strength in the upper limbs and grade 3 in the lower limbs. Upon admission, blood glucose was 3.4 mmol/L, serum total bilirubin was 9.5 mg/dl, and blood gas analysis was normal. During hospitalization, results for NSE (35 ng/ml), blood cell analysis + CRP, urine analysis, stool routine, liver and kidney function tests, electrolytes, blood culture, TORCH tests, procalcitonin, pre-blood transfusion, and Epstein–Barr virus tests were all normal. A spine MRI was unremarkable, while a brain MRI on postnatal day 3 showed symmetrical patchy T1W1 hyperintensity in the bilateral basal ganglia, thalamus, periventricular area, and brainstem (Figure 1). We did genetic testing, which showed a homozygous deletion of exon 7 of the SMN1 gene, with a heterozygous deletion in both parents, confirming a diagnosis of spinal muscular atrophy. Sadly, the child's parents withdrew treatment, and he died of respiratory failure a week later.

## Discussion

In this case, the child was admitted to the hospital with reduced muscle tone as the primary symptom. His brain MRI showed high signal changes in multiple regions on T1WI. Neonatal brain MRI signal changes can have various causes, we excluded the diagnosis of bilirubin encephalopathy and the fact that the child had no postnatal hypoxic-ischemic events, such as asphyxia, intrauterine distress, severe hypoglycemia, or significant infections, making the origin of the MRI changes unclear. Ultimately, a diagnosis of SMA was confirmed through genetic testing.

SMA is the second most common fatal autosomal recessive disease in infancy and is classified into five types based on symptom onset and highest motor milestones achieved (2, 3). Patients with type 0, I, and II are more severe, and they mostly die from respiratory failure, while type III and IV life expectancy are generally not affected (1). Two SMA-related genes, SMN1 and SMN2, are located on chromosome 5, differing by a single nucleotide (C-to-T transition in exon 7); SMN1 produces SMN protein normally, whereas SMN2 produces only 10% of the total SMN protein (1). When a homozygous deletion of SMN1 exon 7 occurs on 5q13, SMN2 alone produces insufficient SMN protein, leading to SMA. Therefore, its severity and prognosis are closely linked to the number of SMN2 copies, with fewer copies indicating a more severe disease.

SMN protein levels in the brain have been found to decrease during development, especially in the fetal and postnatal stages (4), suggesting a critical role for SMN protein in the early stages of brain development. Neuropathological data show that severe forms of SMA affect the brain (5), and reduced brain structural size is observed in mouse models of severe SMA, especially in regions associated with high SMN protein levels, suggesting that high SMN protein levels are required for brain development (6). We speculate that the brain MRI changes may be related to SMA, although few studies have explored this. To further explore the characteristics of MRI changes in the brain of SMA, we reviewed the previously published English literature. Three case-control studies showed abnormal gray matter changes in patients with SMA, and seven individual studies reported a total of 16 patients with different types of SMA. They mention the presence of symmetrical high signal in the white matter, putamen and thalamus in patients with SMA type 0 (7); periventricular posterior horn of the lateral ventricle and bilateral anterolateral thalamus high-signal-intensity lesions in children with SMA type I (8); periventricular high intensity around the posterior horns of lateral ventricles and delayed myelin formation in patients with SMA type II (9) (Table 1).

**Table 1:** Review the literature on brain MRI signal changes in SMA patients.

No.	Ref.	Year	Classification of type/number of cases	The main imaging findings
1	Shen et al. (16)	2024	II/22 III/21	Children with type 2 and type 3 SMA have extensive, multifocal, symmetric gray and white matter changes.
2	Losito et al. (9)	2021	II/1	T1W1: There was an area of high intensity around the posterior horn of the lateral ventricle, delayed myelination, and dysplasia of the corpus callosum
3	de Borba et al. (17)	2020	III/19 lV/6	The cerebellar volume of SMA patients was significantly smaller and lobular gray matter was significantly reduced. (T1-weighted image)
4	Maeda K et al. (7)	2019	0/1	Progressive atrophy was observed in the cerebral cortex, subcortical white matter, thalamus, and basal ganglia, and symmetrical hyperintensities were observed in the white matter, putamen, and thalamus.
5	Mendonca et al. (12)	2019	0/3	There was supratentorial atrophy, thinning of the corpus callosum, widening of the sulci and ventricles, severe reduction of white matter (3/3), and severe atrophy of the hippocampus. In two patients, the putamen and thalamus (lateral and occipital) were detected symmetrically hyperintense on T2-weighted images and FLAIR sequences, with ventricular dilatation.
6	Querin G et al. (18)	2019	III/19 lV/6	Patients with SMA have increased gray matter density in motor and extra-motor areas. (T1-weighted image)
7	Ito et al. (8)	2004	I/1	T2-weighted and FLAIR images showed high signal intensity lesions around the posterior horn of the lateral ventricle and bilateral anterolateral thalamus.
8	Oka et al. (19)	1995	I/1	T1w1-weighted images diffuse brain atrophy with more prominent white matter, with agenesis of the corpus callosum, enlargement of the third ventricle and lateral ventricle, and T2-weighted sequences showed areas of hyperintensity around the posterior horn of the lateral ventricle.
9	Cneude et al. (20)	1999	I/1	Central nervous system (thalamus, cerebellum) lesions, Severe cortical dysplasia.
10	Yohannan et al. (21)	1991	I/8	Seven cases showed generalized cortical atrophy, and one case showed hypoattenuated, non-enhancing areas in the white matter involving both frontal lobes.

Further exploration of the pathogenesis suggests that there is a modifier gene for SMA, the zinc finger protein (ZPR1) gene (10). It can interact with SMN sites to induce neuronal differentiation, stimulate axon growth in motor neuron-like cells, and increase SMN levels (10, 11). We hypothesized that previously reported cerebral atrophy may be related to chronic hypoxia in the brain (12), because defects in ZPR1 in critically ill SMA patients contribute to phrenic nerve axonal loss of function and myelin proliferation, leading to defects in diaphragmatic respiratory function causing respiratory muscle weakness, which ultimately can lead to chronic hypoxia in the brain (10), Hypoxia can cause brain MRI signal changes, and abnormal signals may appear in the basal ganglia, thalamus and surrounding cortex (13). Moreover, the low SMN levels in SMA can lead to motor neuron degeneration, potentially causing insufficient myelin maturation. This could explain white matter atrophy and high-signal intensity in the lateral ventricle and thalamus, which may reflect unmyelinated regions or abnormal myelin development (14). Obviously, these brain MRI changes are unusual, and the brain MRI changes in SMA patients are diverse, and we cannot conclude that the brain MRI changes in SMA patients are specific. The mechanism of brain MRI changes in patients with SMA is not fully understood, and the evidence that ZPR1 deficiency allows hypoxic episodes and reduced SMN protein levels to cause brain MRI signal alterations remains insufficient. However, abnormal MRI signal may indicate the presence of SMA, and clinicians should be alert to the occurrence of such diseases. This paper reports abnormal MRI signal changes in a patient with SMA, suggesting a possible connection to the disease. A limitation of this case is that the child did not undergo SMN2 gene copy number testing, but in combination with the time of onset of the disease and the characteristics of the child, we made a clinical diagnosis of SMN type 0.

SMN1 deletion and SMN2 copy number can be detected by a variety of techniques, with genetic testing serving as the gold standard for diagnosing SMA. Although brain MRI is not required for diagnosis, it can reveal structural brain changes and is a widely available, non-invasive tool that may assist in the diagnostic process. Early treatment has been shown to prevent the most severe forms of SMA, with its effectiveness highly dependent on early administration. Presymptomatic treatment can result in normal or mildly subnormal motor development that would otherwise progress to severe disease. Currently, several approaches are available for early treatment of SMA (15), which are based on the general principle of increasing SMN protein expression. Pharmacological or gene therapies that increase SMN2 expression, antisense oligonucleotide (ASO) -based therapies, and virus-mediated therapies are included (1). ZPR1 upregulates SMN2 protein transcription and promotes SMN protein activation for myelin regeneration, and upregulation of ZPR1 expression to increase SMN levels is a viable therapeutic target for the development of new approaches to SMA treatment (10, 11).

## Conclusion

Brain MRI signal changes of SMA are rare, with high mortality and poor prognosis. So far, SMA can only delay the progression of the disease rather than completely cure it (1), which highlights the difficulty and importance of differential diagnosis of this disease. Genetic testing is the key to the diagnosis of SMA, and cranial MRI may be helpful for the diagnosis. Prenatal diagnosis and newborn screening are the most important prevention options, and early identification and treatment can help improve the prognosis of SMA patients.

## Data Availability

The original contributions presented in the study are included in the article/Supplementary Material, further inquiries can be directed to the corresponding author.
